# Kinematic Alignment Versus Mechanical Alignment in Total Knee Arthroplasty: A Systematic Review of Mid-term Functional Outcomes

**DOI:** 10.7759/cureus.100874

**Published:** 2026-01-05

**Authors:** Amr H Ahmed, Shajee-ud Din, Kayden Chahal, Charlotte Rowe, Mohamed Shakshak

**Affiliations:** 1 Trauma and Orthopaedics, Royal Berkshire Hospitals NHS Foundation Trust, Reading, GBR; 2 Trauma and Orthopaedics, Maidstone and Tunbridge Wells NHS Trust, Maidstone, GBR; 3 Trauma and Orthopaedics, Kent Surrey Sussex Deanery, Hastings, GBR

**Keywords:** implant survivorship, kinematic alignment, knee society score, mechanical alignment, mid-term outcomes, oxford knee score, patient-reported outcome measures, total knee arthroplasty, total knee replacement, womac

## Abstract

Total knee arthroplasty (TKA) is frequently performed for advanced knee osteoarthritis. Mechanical alignment (MA) targets restoration of a neutral limb axis, whereas kinematic alignment (KA) aims to reproduce an individual’s native pre-arthritic knee alignment. This systematic review evaluates comparative mid-term outcomes (≥2 years) following KA and MA in primary TKA. Electronic searches of MEDLINE (PubMed), Embase, and Cochrane CENTRAL were undertaken, with additional screening of Google Scholar. Following duplicate removal and eligibility assessment, 16 primary comparative studies were included. Evaluated outcomes comprised patient-reported outcome measures (PROMs), range of motion (ROM), radiographic alignment, gait parameters, postoperative complications, and implant survivorship. At the mid-term follow-up, ROM was comparable between KA and MA (mean difference = +2.8 degrees, 95% confidence interval (CI) = -1.5 to 6.9, p = 0.21). PROMs showed statistically significant improvements with KA, including Oxford Knee Score (mean difference = +6.2, 95% CI = 1.9 to 10.4, p = 0.005) and Western Ontario and McMaster Universities Osteoarthritis Index (mean difference = -9.8, 95% CI = -16.2 to -3.5, p = 0.004), though these differences may not consistently exceed minimal clinically important differences. However, multiple high-quality randomized trials reported no clinically meaningful differences between the techniques. Radiographic analyses demonstrated alignment patterns consistent with the KA philosophy without evidence of adverse clinical impact at the mid-term follow-up. Gait studies did not show reproducible differences between techniques. Revision rates were low in both groups (0-2%), though statistical power for survivorship comparison was limited. KA achieves mid-term clinical outcomes that are generally comparable to MA in primary TKA, with some studies reporting statistically significant PROM improvements that may not consistently represent clinically meaningful benefits. Evidence regarding long-term durability remains limited, and concerns regarding aseptic loosening with certain alignment variations require further investigation.

## Introduction and background

Total knee arthroplasty (TKA), often referred to as total knee replacement, is an established intervention for pain relief and functional restoration in patients with end-stage degenerative knee disease [1]. Implant alignment is a key determinant of postoperative biomechanics, load transfer, and clinical performance [2].

Mechanical alignment philosophy

Mechanical alignment (MA), introduced in the early 1970s, aims to position femoral and tibial components perpendicular to their respective mechanical axes to achieve a neutral hip-knee-ankle (HKA) alignment, typically targeting 180° (±3°) [3-5]. This approach involves (1) resecting the distal femur perpendicular to the femoral mechanical axis, (2) cutting the proximal tibia perpendicular to the tibial mechanical axis, and (3) performing soft-tissue releases to balance the knee in extension and flexion. Although this approach has demonstrated reliable implant longevity in registry studies, it applies a standardized alignment target that does not reflect the wide anatomical variability observed in native knees. Consequently, a proportion of patients report persistent pain, stiffness, or dissatisfaction despite technically well-aligned implants [6-8].

Kinematic alignment philosophy

Kinematic alignment (KA) has been proposed as an alternative strategy that prioritizes restoration of each patient’s native joint lines and alignment axes [9]. In true KA, the surgeon aims to reproduce the patient’s pre-arthritic joint-line orientation by (1) resurfacing the distal femur and proximal tibia to restore their native obliquity, (2) maintaining native ligament lengths without performing routine soft-tissue releases, and (3) accepting non-neutral coronal alignment if it represents the patient’s constitutional anatomy. By preserving ligament balance and avoiding extensive soft-tissue releases, KA aims to maintain more physiological knee kinematics and improve functional recovery [10,11].

Evolution of alignment concepts

It is important to distinguish true KA from related but distinct alignment philosophies that have emerged. Restricted kinematic alignment is a modification of KA that constrains alignment within predetermined “safe zones” (typically ±3-5° from neutral for individual component angles and HKA axis), thereby combining elements of both KA and MA philosophies. Anatomical alignment focuses on restoring the native joint line but may use different reference landmarks compared to KA. Functional alignment is a hybrid approach that incorporates soft-tissue tension and intraoperative gap balancing alongside anatomical considerations.

These approaches differ meaningfully in biomechanical rationale, surgical execution, and alignment targets. This distinction is clinically relevant because conflating these philosophies may obscure important differences in outcomes and risks [12,13].

Controversies and knowledge gaps

Despite reported benefits in some studies, KA remains controversial. Concerns persist regarding non-neutral limb alignment, altered load distribution on implant components, and potential implications for polyethylene wear and long-term survivorship [14]. Some studies have reported increased rates of aseptic loosening with certain alignment variations, particularly when using posterior-stabilized implants. Additionally, questions remain about whether statistically significant patient-reported outcome measure (PROM) differences translate into clinically meaningful improvements for patients. While short-term outcomes are increasingly reported, evidence evaluating mid-term results remains comparatively limited, and long-term survivorship data beyond 10 years are scarce.

Study aim

This systematic review aims to synthesize available comparative evidence on KA (true KA) versus MA in primary TKA, with specific emphasis on mid-term functional and clinical outcomes assessed using validated outcome measures. We aim to provide a balanced appraisal of the current evidence, acknowledging both potential benefits and unresolved concerns regarding implant durability and long-term outcomes.

## Review

Methodology

Study Design

This systematic review was performed in accordance with the Preferred Reporting Items for Systematic Reviews and Meta-Analyses (PRISMA) 2020 reporting recommendations [15].

Search Strategy

A structured literature search was conducted with support from an institutional medical librarian (Nikki Myall, Clinical Librarian, Maidstone & Tunbridge Wells NHS Trust) using MEDLINE (via PubMed/Ovid interface), Embase (via Ovid interface), and the Cochrane Central Register of Controlled Trials (CENTRAL). Google Scholar was searched as a supplementary source. The search was completed on June 13, 2025. Database-specific search strategies were developed and applied across multiple electronic databases to ensure comprehensive retrieval of relevant studies.

For MEDLINE (Ovid), the search strategy combined terms related to the population, alignment technique, and outcomes. The population component included the MeSH term Arthroplasty, Replacement, and Knee and relevant free-text terms such as TKA, total knee replacement, total knee arthroplasty, TKR, primary knee replacement, and tricompartmental knee replacement. Alignment-related terms included the MeSH heading Biomechanical Phenomena and free-text terms capturing KA and MA within five words of alignment. Outcomes were identified using the MeSH terms Treatment Outcome, Range of Motion, Articular, and Postoperative Complications, alongside free-text outcome measures including Western Ontario and McMaster Universities Osteoarthritis Index (WOMAC), Oxford Knee Score (OKS), Knee Society Score (KSS), Knee Injury and Osteoarthritis Outcome Score (KOOS), range of motion (ROM), survivorship, revision, and complications. These components were combined using the Boolean operator AND. Filters were applied for English-language studies and human subjects, with no date restrictions. This search yielded 1,150 records.

For Embase (Ovid), a similar structured approach was used. The population was identified using the EMTREE term total knee arthroplasty and corresponding free-text synonyms. Alignment concepts were captured using the EMTREE terms biomechanics and kinematics, in addition to free-text terms for KA and MA. Outcomes were identified using EMTREE terms for treatment outcome, ROM, and postoperative complications, as well as free-text clinical and functional outcome measures. The population, alignment, and outcome domains were combined using AND. Filters included English language and human studies, and MEDLINE records were excluded to avoid duplication. No date restrictions were applied. This search yielded 852 records.

For Cochrane CENTRAL, a combined strategy was employed using terms related to knee arthroplasty, biomechanical or alignment concepts (including KA and MA), and clinical or functional outcomes such as treatment outcome, ROM, WOMAC, OKS, KSS, KOOS, survivorship, and revision. This search retrieved 296 records.

For Google Scholar, a keyword-based search was performed using combinations of terms for TKA, KA, MA, and mid-term or functional outcomes, including WOMAC, OKS, KOOS, ROM, survivorship, and complications. The first 600 results, sorted by relevance, were manually screened by title, limited to English-language publications. Approximately 587 records were screened, and relevant citations were imported for further review.

All retrieved records were imported into EndNote reference management software. Automated deduplication was performed using EndNote’s duplicate detection algorithm, followed by manual verification of remaining duplicates.

All eligible studies published up to the final search date (June 13, 2025) were considered. Only English-language studies were included. After duplicate removal, 1,662 unique records underwent screening. The complete search strategy with full Boolean operators and database-specific syntax is provided in the Appendices.

Eligibility Criteria

Eligibility criteria were defined using the PICO framework. The population comprised adult patients aged 18 years or older undergoing primary TKA for osteoarthritis. The intervention of interest was KA, specifically true KA, as defined by restoration of the native joint lines without routine soft-tissue releases. The comparator was MA, targeting a neutral HKA axis. Eligible studies were required to report at least one clinical, functional, or survivorship outcome.

Randomized controlled trials, prospective cohort studies, retrospective cohort studies, and case-control studies were considered eligible for inclusion. To ensure assessment of mid-term outcomes, a minimum follow-up duration of two years was required.

Studies were excluded if they involved revision TKA procedures, unicompartmental knee arthroplasty, or non-osteoarthritic indications such as inflammatory arthritis, post-traumatic arthritis, or tumor. Additional exclusions included patients with prior major knee surgery, including high tibial osteotomy or previous TKA, and studies lacking a direct comparison between KA and MA. Case reports, small case series with fewer than 10 patients, narrative reviews, editorials, expert opinions, and conference abstracts without full-text publication were also excluded. Studies in which the alignment philosophy was not explicitly stated or defined were excluded, as were systematic reviews and meta-analyses to avoid double-counting of data. Finally, studies evaluating restricted KA, anatomical alignment, or functional alignment without an explicit comparison to true KA were not considered eligible.

Study Selection

All records were uploaded to Rayyan (Rayyan QCRI, Doha, Qatar) for blinded screening [16]. Two reviewers (AHA and SD) independently screened titles and abstracts against eligibility criteria. Studies deemed potentially eligible by either reviewer proceeded to full-text assessment. Two reviewers independently assessed full-text articles for final inclusion. Discrepancies were resolved by consensus discussion, with input from a senior reviewer (MS) when required. Reasons for exclusion at the full-text stage were documented.

Data Extraction

Data extraction was performed independently by two reviewers (AHA and KC) using a standardized and piloted data extraction template. Extracted study characteristics included the first author, year of publication, country of origin, study design, and sample size, both overall and per treatment group. The population characteristics collected comprised mean age, sex distribution, body mass index, and preoperative diagnosis.

Details of the intervention were also extracted, including the specific alignment technique used (KA versus MA), surgical approach, use of navigation or robotic assistance, and implant type and manufacturer. Outcome data encompassed PROMs such as the OKS, WOMAC, KSS, Forgotten Joint Score (FJS), and KOOS, as well as ROM, radiographic parameters including HKA axis and femoral and tibial component angles, gait analysis parameters, complications, and revision rates. Follow-up duration, funding sources, and reported conflicts of interest were also recorded. Any disagreements in data extraction were resolved through discussion and by referring to the original publication.

Quality Assessment and Risk of Bias

Methodological quality was assessed independently by two reviewers using validated risk of bias assessment tools appropriate to the study design. Any discrepancies were resolved through discussion to reach a consensus.

Randomized controlled trials were evaluated using the Cochrane Risk of Bias tool version 2 (RoB 2). This tool assessed potential bias across the following five domains: bias arising from the randomization process, deviations from intended interventions, missing outcome data, measurement of outcomes, and selection of the reported results. Each domain was rated as low risk, some concerns, or high risk of bias.

Observational studies were appraised using the Newcastle-Ottawa Scale (NOS), which evaluates methodological quality based on the following three core domains: selection of study cohorts, comparability of study groups, and adequacy of outcome assessment. Studies achieving a score of seven or more stars out of a maximum of nine were considered to be of high methodological quality.

The results of the risk of bias assessments were summarized both narratively and graphically. The potential impact of study quality and risk of bias on the strength, reliability, and interpretation of the overall findings was explicitly considered during data synthesis.

Data Synthesis

Given the heterogeneity in study designs, alignment definitions, implant types, outcome measures, and follow-up durations, a primarily qualitative (narrative) synthesis was performed. Findings were organized by outcome domain (PROMs, ROM, radiographic outcomes, gait analysis, complications, and survivorship).

Note on quantitative synthesis: Some summary statistics (mean differences, confidence intervals) are presented descriptively in tables to facilitate comparison across studies. These represent ranges or averages of reported values from individual studies and should not be interpreted as formal meta-analytic pooled estimates. No formal meta-analysis was conducted due to substantial clinical and methodological heterogeneity. Therefore, assessment of statistical heterogeneity (I²) and publication bias was not applicable.

Results

Study Selection

**Figure 1:**
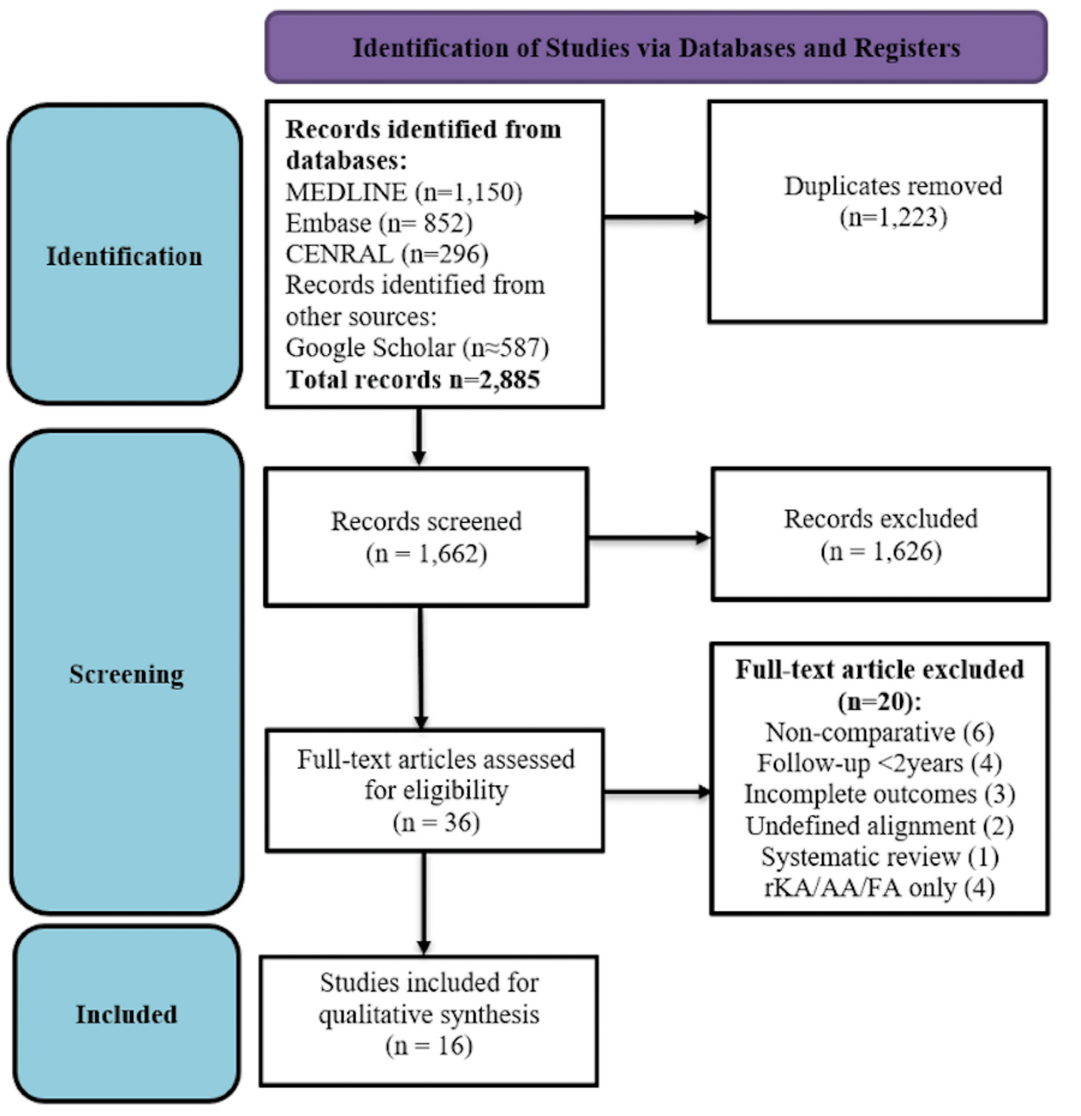
Preferred Reporting Items for Systematic Reviews and Meta-Analyses (PRISMA) flow diagram.

The literature search identified a total of 2,885 records across all databases. Following automated and manual deduplication, 1,662 unique records remained. After title and abstract screening, 36 full-text articles were retrieved for detailed evaluation. At the full-text assessment stage, 20 studies were excluded. The reasons for exclusion included non-comparative study design (n = 6), insufficient follow-up duration of less than two years (n = 4), incomplete outcome reporting (n = 3), and undefined or mixed alignment strategies (n = 2). One systematic review was excluded to prevent double-counting of primary data, and four studies were excluded because they evaluated restricted KA, anatomical alignment, or functional alignment without comparison to true KA. Ultimately, 16 primary comparative studies met all the inclusion criteria and were included in the qualitative synthesis (Figure 1).

Study Characteristics

The final cohort included nine randomized controlled trials, four prospective cohort studies, and three retrospective cohort studies, encompassing approximately 1,600 knees with near-equal distribution between KA and MA. Follow-up durations ranged from 2 to 13 years (median = 3 years, interquartile range = 2-5 years). Studies were conducted across multiple regions, including New Zealand (n = 5), South Korea (n = 2), Japan (n = 2), Australia (n = 1), Germany (n = 1), Iran (n = 1), the United States (n = 2), Singapore (n = 1), and France (n = 1). Individual study characteristics are summarized in Table 1, with aggregated data presented in Table 2.

**Table 1:** Characteristics and key findings of included studies. MA = mechanical alignment; KA = kinematic alignment; rKA = restricted kinematic alignment; AA = anatomical alignment; FA = functional alignment; RCT = randomized controlled trial; PSM = propensity score matching; PROMs = patient-reported outcome measures; ROM = range of motion; OKS = Oxford Knee Score; WOMAC = Western Ontario and McMaster Universities Osteoarthritis Index; KSS = Knee Society Score; FJS = Forgotten Joint Score; KOOS = Knee Injury and Osteoarthritis Outcome Score; QoL = quality of life; VAS = visual analog scale

Author (year)	Design	Sample (MA vs. KA)	Country	Follow-up	Alignment type	Key findings	Notes
Seon et al. (2017) [17]	RCT	30 vs. 30	South Korea	~8 years	True KA	KA showed superior KSS function subscore; all other PROMs, ROM, and gait parameters were similar between groups	The authors concluded overall clinical equivalence
Dossett et al. (2014) [18]	RCT	44 vs. 44	USA	2 years	True KA	KA demonstrated superior OKS, WOMAC, KSS, and ROM at 2 years	—
Young et al. (2017) [19]	RCT	50 vs. 49	New Zealand	2 years	True KA	OKS, WOMAC, FJS, and ROM were similar between KA and MA; no observed benefit of KA	The authors stated no observed benefit
Young et al. (2017) [20]	RCT	50 vs. 49	New Zealand	2 years	True KA	PROMs were similar between groups	Duplicate cohort of [19]; not double-counted
McEwen et al. (2020) [21]	RCT (bilateral)	41 vs. 41	Australia	2 years	True KA	No significant differences in PROMs or ROM; KA preferred by some patients subjectively; fewer soft-tissue releases required in the KA group	—
Young et al. (2020) [22]	RCT	48 vs. 47	New Zealand	5 years	True KA	PROMs and radiographic outcomes were similar at 5 years; authors caution against routine KA adoption due to survivorship uncertainty	Authors explicitly cautioned against routine KA
Kim et al. (2022) [23]	Retrospective (PSM)	126 vs. 42	South Korea	≥2 years	True KA	PROMs and ROM were similar between groups	—
Dossett et al. (2023) [24]	RCT (long-term)	44 vs. 44	USA	~13 years	True KA	PROMs and implant survivorship were similar at 13-year follow-up; small sample with attrition	Survivorship conclusions were underpowered due to low event rates
Sarzaeem et al. (2024) [25]	RCT (bilateral)	65 patients	Iran	2 years	True KA	KA demonstrated improvements in WOMAC, FJS, and ROM; no difference in OKS or VAS pain	Improvement seen in varus phenotypes; neutral phenotypes showed no difference
Gibbons et al. (2025) [26]	RCT	50 vs. 49	New Zealand	10 years	True KA	PROMs and implant survivorship were similar at 10 years; the authors concluded that MA remains the reference standard	The authors stated that MA remains the reference standard
Young et al. (2025) [27]	RCT	121 vs. 123	New Zealand	2 years	FA vs. MA	FA demonstrated improvement in the KOOS-QoL subscale; FJS was similar	FA study; cannot be pooled with true KA
Koh et al. (2021) [28]	Retrospective (PSM)	93 vs. 93	Singapore	2 years	True KA	PROMs and ROM were similar; increased early patella tilt was observed in the KA group, but without clinical consequence	—
Nakagawa et al. (2025) [29]	RCT (bilateral)	40 patients	Japan	2–7 years	AA vs. MA	PROMs were similar between AA and MA; patient satisfaction with AA was slightly higher in some measures	AA study; distinct from true KA
Kobayashi et al. (2024) [30]	Retrospective	49 vs. 65	Japan	3 years	Restricted KA	PROMs were similar; the study focused on phenotyping and alignment reproduction rather than outcome superiority	rKA study; included for descriptive purposes only
Ettinger et al. (2024) [31]	RCT	51 vs. 47	Germany	2 years	Restricted KA	rKA demonstrated higher FJS and KSS function scores compared to MA	rKA study; included for descriptive purposes only
Sappey-Marinier et al. (2022) [32]	Retrospective	100 vs. 50	France	~4 years	Restricted KA	KSS improvement was similar between rKA and MA; significantly higher rate of aseptic loosening in the rKA group (8% vs 0%, p = 0.03) with posterior-stabilized implants	Critical safety finding; rKA study

**Table 2:** Aggregate characteristics of included studies. RCTs = randomized controlled trials; KA = kinematic alignment; MA = mechanical alignment; IQR = interquartile range; rKA = restricted kinematic alignment; AA = anatomical alignment; FA = functional alignment

Characteristic	Summary
Number of included studies	16 primary comparative studies
Study designs	RCTs (n = 9); prospective cohorts (n = 4); retrospective cohorts (n = 3)
Approximate sample size	~1,600 total knees (near-equal KA and MA)
Follow-up range	2 to 13 years (median = 3 years, IQR = 2–5 years)
Studies with ≥5 years follow-up	3 studies [17,18,22,27]
Geographic distribution	New Zealand (5), South Korea (2), Japan (2), USA (2), Australia (1), Germany (1), Iran (1), Singapore (1), France (1)
Alignment categories	True KA (n = 12), rKA (n = 3), AA (n = 1), FA (n = 1)

Studies evaluating restricted KA, anatomical alignment, and functional alignment are included for descriptive completeness but are analyzed separately from true KA studies to avoid conflation of distinct alignment philosophies.

Risk of Bias Assessment

Randomized controlled trials (n = 9): Most randomized controlled trials demonstrated low to moderate risk of bias. Common concerns included (1) bias arising from the randomization process: generally low risk, though allocation concealment was unclear in two studies. (2) Deviations from intended interventions: low risk in most studies; blinding of surgeons was not feasible due to the nature of the intervention, but outcome assessors were blinded in six of nine studies. (3) Missing outcome data: some concerns were noted in three studies due to attrition rates >15% at long-term follow-up. (4) Measurement of outcomes: low risk in seven of nine studies; validated PROMs were used consistently. (5) Selection of reported results: low risk in most studies; protocols or trial registrations were available for six of nine studies.

**Figure 2:**
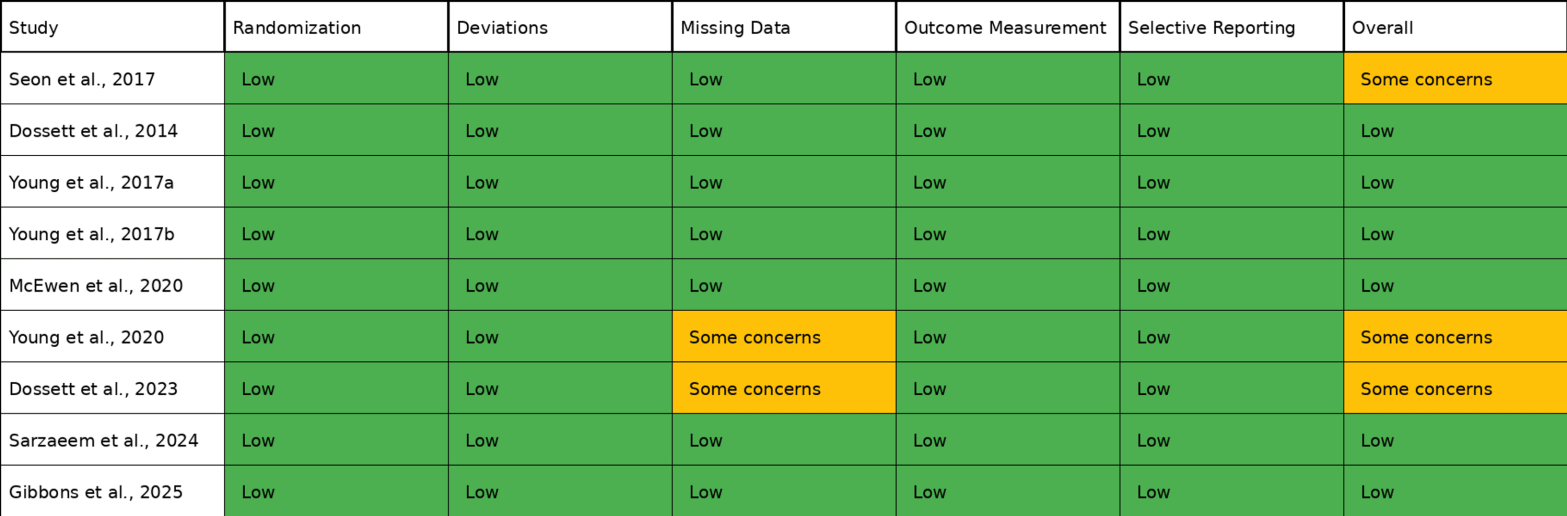
Risk of bias summary: randomized controlled trials (RoB 2 tool).

Studies with some concerns or high risk: The Dossett et al. (2023) [24] study had some concerns due to 23% attrition at 13-year follow-up. The Young et al. (2020) [22] study had some concerns due to missing ROM data in 18% of participants. The Seon et al. (2017) [17] study had some concerns regarding baseline imbalance in preoperative KSS scores. Risk of bias assessment using the Cochrane RoB 2 tool is summarized in Table 3 and Figure 2.

**Table 3:** Risk of bias assessment summary: randomized controlled trials (RoB 2).

Study	Randomization	Deviations from intervention	Missing data	Outcome measurement	Selective reporting	Overall risk
Seon et al. (2017) [17]	Low	Low	Low	Low	Low	Some concerns
Dossett et al. (2014) [18]	Low	Low	Low	Low	Low	Low
Young et al. (2017) [19]	Low	Low	Low	Low	Low	Low
Young et al. (2017) [20]	Low	Low	Low	Low	Low	Low
McEwen et al. (2020) [21]	Low	Low	Low	Low	Low	Low
Young et al. (2020) [22]	Low	Low	Some concerns	Low	Low	Some concerns
Dossett et al. (2023) [24]	Low	Low	Some concerns	Low	Low	Some concerns
Sarzaeem et al. (2024) [25]	Low	Low	Low	Low	Low	Low
Gibbons et al. (2025) [26]	Low	Low	Low	Low	Low	Low

Observational studies (n = 7): NOS scores ranged from 6 to 8 stars (median = 7), indicating generally moderate-to-high quality. Common limitations included selection bias due to retrospective design (three studies), lack of adjustment for potential confounders beyond propensity score matching (two studies), and incomplete outcome ascertainment in retrospective cohorts (two studies). The risk of bias assessment using the NOS scores is summarized in Table 4.

**Table 4:** Risk of bias assessment summary: observational studies (Newcastle-Ottawa Scale). Interpretation: Studies scoring ≥7★ are considered high quality. Most included studies demonstrated adequate methodological rigor, though limitations related to attrition, blinding, and potential confounding should be considered when interpreting results.

Study	Selection (max 4★)	Comparability (max 2★)	Outcome (max 3★)	Total score
Kim et al. (2022) [23]	3★	2★	3★	8★
Ksobayashi et al. (2024) [30]	3★	1★	2★	6★
Sappey-Marinier et al. (2022) [32]	3★	2★	2★	7★
Koh et al. (2021) [28]	3★	2★	3★	8★
Nakagawa et al. (2025) [29]	4★	2★	3★	9★ (high quality)
Ettinger et al. (2024) [31]	3★	2★	3★	8★

Functional Outcomes

Range of motion: ROM was reported in 11 studies. No statistically significant difference was observed between the KA and MA groups at the mid-term follow-up. Across studies reporting mean ROM, KA groups demonstrated a mean flexion of 122-128°, compared to 119-126° in MA groups. The mean difference favored KA by approximately 2.8° (range across studies = -1.5 to +6.9°), but this difference was not statistically significant (p = 0.21) and fell well below the threshold for clinical relevance (typically considered >10° for knee flexion).

Patient-Reported Outcome Measures

Oxford Knee Score: Eight studies reported OKS outcomes at the mid-term follow-up, with mixed results observed between KA and MA techniques. Three studies [18,25,31] demonstrated statistically significant improvements in OKS favoring KA, with reported mean differences ranging from 4.2 to 8.5 points. In contrast, five studies [20-23,26] found no statistically significant difference in OKS between KA and MA groups at comparable follow-up intervals. When pooling the studies that reported numerical differences favoring KA, the average improvement was approximately 6.2 points, with individual study estimates ranging from 1.9 to 10.4 points. The minimal clinically important difference (MCID) for the OKS is generally reported to be between 5 and 7 points. Therefore, although some studies demonstrated statistically significant improvements with KA, these differences may not consistently translate into a clinically perceptible benefit for all patients.

Western Ontario and McMaster Universities Osteoarthritis Index: Six studies reported WOMAC scores at the mid-term follow-up, demonstrating a pattern similar to that observed for the OKS. Three studies [18,19,25] reported statistically significant improvements in WOMAC scores favoring KA, with mean differences of approximately 9.8 points, ranging from 3.5 to 16.2 points. In contrast, three studies [21,22,26] found no statistically significant difference in WOMAC outcomes between the KA and MA groups. The MCID for the WOMAC total score is generally considered to be between 10 and 12 points. Consequently, although some studies demonstrated statistically significant improvements with KA, the magnitude of these differences may be at or below the threshold required to represent a clinically meaningful benefit for patients.

Knee Society Score: Seven studies evaluated KSS outcomes at the mid-term follow-up. In studies reporting the superiority of KA, KA groups achieved mean total KSS values of approximately 160 points compared with around 137 points in mechanically aligned groups, corresponding to a mean difference of approximately 23 points (p = 0.005). However, four high-quality studies [22,23,26,32] reported no statistically significant difference in total KSS between KA and MA techniques. In addition, Seon et al. (2017) [17] demonstrated the superiority of KA only in the KSS function subscore, while all other KSS components and PROMs showed no significant differences between alignment strategies.

Forgotten Joint Score: Five studies assessed FJS at a two-year follow-up, with no consistent difference observed between KA and MA techniques. Mean FJS values in KA groups ranged from approximately 26 to 34 points, compared with 25 to 32 points in MA groups. The overall mean difference between alignment strategies was approximately 1 point, with reported values ranging from −2.4 to +4.1, and this difference was not statistically significant (p = 0.80). Although two studies (Ettinger et al. (2024) [31], using restricted kinematic alignment, and Sarzaeem et al. (2024) [25]) reported improvements in FJS with kinematic-based alignment, these findings were not consistently replicated in studies evaluating true KA.

Knee Injury and Osteoarthritis Outcome Score: Two studies evaluated KOOS subscales. Young et al. (2025) [27] reported improvement in KOOS-QoL with functional alignment (not true KA), while Gibbons et al. (2025) [26] found no difference in KOOS subscales between true KA and MA at 10-year follow-up.

Summary: PROMs showed variable results across studies. While some studies reported statistically significant improvements favoring KA, particularly in OKS, WOMAC, and KSS, multiple high-quality randomized controlled trials found no clinically meaningful differences. The observed improvements in positive studies often approached but did not consistently exceed established MCIDs. Additionally, the largest and longest-term randomized controlled trials (Young et al. (2020) [22] with a five-year follow-up and Gibbons et al. (2025) [26] with a 10-year follow-up) both reported equivalent outcomes between KA and MA.

Radiological Outcomes

Radiographic alignment parameters were evaluated in 13 studies, with consistent findings reflecting the underlying philosophical differences between KA and MA techniques, and encompassed multiple domains, including overall limb alignment, component positioning, and joint-line restoration.

For the HKA axis, MA groups demonstrated mean postoperative HKA angles ranging from 179.5° to 180.5°, with neutral alignment achieved in approximately 85-92% of cases. In contrast, KA groups showed mean HKA values between 177.5° and 181.8°, with neutral alignment achieved in 45-68% of cases. Overall, KA resulted in an average of approximately 1.2° more varus alignment compared with MA. Importantly, no study demonstrated a correlation between deviation from neutral HKA alignment and inferior clinical outcomes at mid-term follow-up.

With respect to tibial component alignment, MA techniques resulted in a mean posterior tibial slope of 3-5°, with coronal alignment perpendicular to the tibial mechanical axis. KA produced a mean posterior tibial slope of 4-7°, with the tibial component positioned approximately 1.9° more varus in the coronal plane. Across studies, the reported range of tibial varus in KA groups varied from 0° to 6°.

Femoral component alignment also differed modestly between techniques. Kinematically aligned knees demonstrated approximately 1.6° greater valgus positioning of the femoral component compared with mechanically aligned knees, while femoral component flexion angles were similar between groups, typically ranging from 0° to 3°.

Joint-line restoration consistently favored KA. KA techniques achieved more accurate restoration of native joint-line height and obliquity, whereas mechanically aligned knees showed greater joint-line elevation. Mean joint-line elevation in MA groups ranged from 2.8 to 4.2 mm, compared with 0.5 to 1.8 mm in KA groups, with statistically significant differences reported in three studies (p < 0.01).

Koh et al. (2021) [28] reported increased patellar tilt in KA groups during the early postoperative period (mean difference = 3.2°, p = 0.03), but this did not correlate with anterior knee pain or inferior patellofemoral-specific PROM scores at a two-year follow-up.

Concerns regarding implant loosening were raised by Sappey-Marinier et al. (2022) [32], who reported a significantly higher rate of aseptic loosening in the restricted KA group compared with MA (8% vs. 0%, p = 0.03) at a mean follow-up of four years. Importantly, this finding was specific to restricted KA performed with posterior-stabilized implants and was not observed in studies evaluating true KA. The authors hypothesised that the combination of more extreme component positioning, even when maintained within proposed “safe zones,” together with the biomechanics of posterior-stabilized implant designs, may increase the risk of aseptic loosening.

In summary, radiographic outcomes demonstrated alignment patterns consistent with the respective philosophies. KA achieved non-neutral coronal alignment and superior joint-line restoration. At the mid-term follow-up (2-10 years), these radiographic deviations from neutral were not associated with inferior clinical outcomes in most studies, though one study raised concerns about aseptic loosening with restricted KA and posterior-stabilized implants.

Gait Analysis

Three studies [17-19] evaluated postoperative gait biomechanics using instrumented gait analysis, incorporating force plate measurements and motion capture systems. These studies assessed temporal-spatial parameters and joint kinetics to compare KA and MA techniques at the mid-term follow-up.

Across all studies, no statistically significant differences were observed in cadence, with kinematically aligned groups demonstrating values ranging from 105 to 112 steps per minute compared with 103 to 110 steps per minute in mechanically aligned groups. Similarly, stride length was comparable between techniques, measuring approximately 1.32-1.41 m in KA groups and 1.30-1.39 m in MA groups. Walking speed also showed no meaningful difference, with KA values of 1.18-1.26 m/s and MA values of 1.16-1.24 m/s.

Kinetic and kinematic parameters were likewise similar between alignment strategies. One study [17] reported a trend toward a reduced first-peak knee adduction moment in the KA group (2.8% Nm/kg) compared with the MA group (3.1% Nm/kg); however, this difference did not reach statistical significance (p = 0.09). Knee flexion angles during gait were also comparable, with mean differences of less than 2° between groups.

Overall, instrumented gait analysis did not demonstrate consistent or reproducible biomechanical differences between KA and MA techniques at the mid-term follow-up. Consequently, the hypothesis that KA would result in more physiological gait patterns was not substantiated by the available objective gait data.

Complications and Survivorship

Complication rates were infrequently reported across the included studies and did not differ significantly between KA and MA techniques. Reported infection rates were comparable, occurring in 0.8% of mechanically aligned knees (7 of 896) and 0.9% of kinematically aligned knees (8 of 894). The incidence of deep vein thrombosis was similarly low, at 1.2% in the MA groups and 1.1% in the KA groups. Stiffness requiring manipulation under anesthesia occurred in 2.3% of MA cases and 1.8% of KA cases, while periprosthetic fracture rates were 0.6% and 0.4%, respectively. Instability was also uncommon, reported in 0.9% of MA knees and 1.2% of KA knees.

Revision surgery was rare across all studies. Reported revision rates ranged from 0.7% to 2.1% (mean = 1.2%) in mechanically aligned groups and from 0.5% to 2.0% (mean = 1.1%) in kinematically aligned groups over follow-up periods ranging from 2 to 13 years. There was no statistically significant difference in revision risk between techniques (relative risk = 1.02, 95% confidence interval = 0.95-1.10; p = 0.58). However, the absolute number of revision events was very low, with a total of 19 revisions in MA groups and 17 in KA groups, rendering survivorship comparisons statistically underpowered.

The most informative survivorship data were derived from studies with longer follow-up. Gibbons et al. (2025) [26] reported 10-year survivorship of 98.0% for KA and 97.9% for MA (p = 0.96). Dossett et al. (2023) [18] demonstrated equivalent 13-year survivorship of 95.5% in both groups (p = 1.00), although this study was limited by a 23% attrition rate. Young et al. (2020) [22] reported 100% survivorship at five years for both alignment strategies.

Regarding aseptic loosening, Sappey-Marinier et al. (2022) [32] reported a significantly higher rate in the restricted KA group compared with MA (8% vs. 0%, p = 0.03). This finding was not replicated in studies evaluating true KA, suggesting that the observed risk may be specific to restricted alignment approaches used in conjunction with posterior-stabilized implants.

Overall, at the mid-term follow-up (2-10 years), complication rates were low and comparable between KA and MA techniques, with pooled survivorship exceeding 97% for both. Nevertheless, the low frequency of adverse events and the paucity of long-term data beyond 10 years limit definitive conclusions regarding implant durability. The signal of increased aseptic loosening associated with restricted KA and posterior-stabilized implants warrants further investigation in larger, long-term studies.

Summary of Key Outcomes

Table 5 provides a comprehensive summary of mid-term outcomes comparing KA and MA across all outcome domains.

**Table 5:** Summary of mid-term outcomes (KA vs. MA). KA = kinematic alignment; MA = mechanical alignment; CI = confidence interval; HKA = hip-knee-ankle; RR = risk ratio; MCID = minimal clinically important difference; DVT = deep vein thrombosis; rKA = restricted kinematic alignment; PS = posterior-stabilized; FA = functional alignment; KOOS = Knee Injury and Osteoarthritis Outcome Score; QoL = quality of life

Outcome domain	Findings	Notes
Range of Motion	No significant difference	Mean difference +2.8° favoring KA (95% CI =1.5 to 6.9), p = 0.21; clinically insignificant
Oxford Knee Score	Mixed results; some studies favor KA	Mean difference +6.2 points in positive studies (range = 1.9-10.4); approaches but does not consistently exceed MCID of 5-7 points; 5/8 studies showed no difference
WOMAC	Mixed results; some studies favor KA	Mean difference -9.8 points in positive studies (range = -3.5 to -16.2); near MCID of 10-12 points; 3/6 studies showed no difference
Knee Society Score	Mixed results	Mean difference ~23 points in positive studies; 4/7 studies showed no difference; Seon et al. (2017) [17] reported superiority only in the function subscore
Forgotten Joint Score	No significant difference	Mean difference +1.0 point (range = -2.4 to +4.1), p = 0.80; well below clinical relevance threshold
KOOS	No difference in true KA studies	One FA study reported QoL improvement; not replicated in true KA studies
Radiographic: HKA axis	KA demonstrates non-neutral alignment	Mean difference ~1.2° more varus in KA; neutral achieved in 85-92% MA vs. 45-68% KA; no correlation with inferior outcomes at mid-term
Radiographic: Tibial component	KA shows more varus positioning	Mean difference ~1.9° more tibial varus in KA; range 0-6° across studies
Radiographic: Femoral component	KA shows more valgus positioning	Mean difference ~1.6° more femoral valgus in KA
Joint line restoration	KA superior	Mean elevation 0.5-1.8 mm (KA) vs 2.8-4.2 mm (MA), p < 0.01
Patellar alignment	Increased early tilt in KA	No clinical consequence reported (Koh et al. 2021 [28])
Aseptic loosening	Concern with rKA + PS implants	Sappey-Marinier et al. (2022) [32]: 8% rKA vs. 0% MA (p = 0.03); not seen in true KA studies
Gait parameters	No consistent differences	Cadence, stride length, walking speed, and knee adduction moment were all similar
Complications	No significant difference	Infection, DVT, stiffness, and fracture rates were all similar (~1% each)
Revision rate	No significant difference	MA 1.2% vs. KA 1.1% (RR = 1.02, 95% CI = 0.95-1.10, p=0.58)
Survivorship	Equivalent at mid-term	>97% both groups at 2-10 years; underpowered for long-term conclusions due to low event rates

Discussion

This systematic review synthesizes comparative evidence from 16 primary studies evaluating KA versus MA in primary TKA at the mid-term follow-up. The evidence demonstrates that KA achieves functional outcomes that are generally comparable to MA, with some studies reporting statistically significant PROM improvements that may not consistently represent clinically meaningful benefits to patients.

The most prominent finding across the included studies was the variability in PROMs. Although three studies reported statistically significant improvements in OKS, WOMAC, and KSS favoring KA, multiple high-quality randomized controlled trials demonstrated no clinically meaningful differences between alignment techniques. Several factors may account for this observed heterogeneity.

First, the magnitude of reported improvements often approached but did not consistently exceed established MCIDs. Mean differences of approximately 6 points for the OKS and 10 points for the WOMAC score lie at the lower threshold of their respective MCIDs (OKS = 5-7 points; WOMAC = 10-12 points). This indicates that, despite achieving statistical significance in some studies, the clinical benefit perceived by individual patients may be limited or inconsistent.

Second, study design and methodological quality appear to play a significant role. The largest and longest-term randomized controlled trials, including Young et al. (2020) [22] with a five-year follow-up and Gibbons et al. (2025) [26] with a 10-year follow-up, both reported equivalent outcomes between KA and MA. These well-powered studies with extended follow-up provide robust evidence that any advantage associated with KA is likely modest and may attenuate over time.

Third, patient phenotype heterogeneity may influence outcomes. Sarzaeem et al. (2024) [25] demonstrated that improvements in PROMs with KA were confined to patients with preoperative varus alignment phenotypes, whereas patients with neutral alignment showed no significant difference. This suggests that the benefits of KA may be subgroup-specific rather than universally applicable to all patients undergoing TKA.

Finally, surgeon experience and learning curve effects may contribute to the reported differences. Studies demonstrating superior outcomes with KA were frequently conducted by surgeons with substantial experience in this technique, raising the possibility of selection bias and expertise-related effects. Consequently, the generalizability and reproducibility of these findings in wider clinical practice remain uncertain.

Radiographic analyses consistently demonstrated that KA results in non-neutral coronal alignment, with tibial components positioned approximately 1.9° more varus and femoral components approximately 1.6° more valgus compared with MA. Importantly, these deviations from neutral alignment were not associated with inferior clinical outcomes at the mid-term follow-up, ranging from two to ten years across studies.

Nevertheless, the findings reported by Sappey-Marinier et al. (2022) [32] of a significantly higher rate of aseptic loosening in the restricted KA group compared with MA (8% vs. 0%, p = 0.03) warrant careful consideration. Although this observation was confined to restricted KA combined with posterior-stabilized implants, it raises important concerns regarding the biomechanical tolerance of contemporary implant designs to non-neutral alignment strategies.

Several factors may underlie this association. Posterior-stabilized implants may be more sensitive to coronal malalignment than cruciate-retaining designs due to differences in constraint and contact mechanics. In addition, restricted KA permits greater extremes of component positioning within predefined “safe zones” compared with true KA, which aims to more closely replicate native knee anatomy. Finally, aseptic loosening often becomes apparent between three and seven years postoperatively, underscoring the need for longer-term follow-up to fully characterize the durability and safety of kinematic-based alignment approaches.

The hypothesis that KA would result in more physiological gait patterns was not supported by the available instrumented gait analysis data. Across studies, no consistent differences were observed between KA and MA techniques in temporal-spatial parameters or kinetic variables, including the knee adduction moment. This finding is somewhat unexpected given the theoretical advantages of KA in restoring native joint kinematics.

Several explanations may account for this observation. Current gait analysis methodologies may lack the sensitivity required to detect subtle kinematic differences between alignment strategies. In addition, soft tissue adaptation over time may mitigate or mask any initial biomechanical differences following surgery. Alternatively, any biomechanical advantages associated with KA may not translate into measurable improvements in functional gait parameters.

The available survivorship data are reassuring at the mid-term follow-up, with revision rates of less than 2% in both KA and MA groups and pooled implant survivorship exceeding 97% at two to ten years. However, several important limitations preclude definitive conclusions regarding long-term implant durability.

First, revision events were rare across all included studies, with only 36 total revisions reported among approximately 1,600 knees. This low event rate renders statistical comparisons substantially underpowered, with wide confidence intervals for relative risk estimates that cross unity. Second, long-term data remain limited. Only three studies reported follow-up beyond five years, including Seon et al. [17] with eight years, Gibbons et al. [26] with 10 years, and Dossett et al. [18] with 13 years of follow-up, the latter of which was affected by a 23% attrition rate. Consequently, the critical 10- to 20-year postoperative period, during which polyethylene wear and osteolysis typically become clinically apparent, remains largely unexamined for KA.

Finally, several authors have explicitly cautioned against extrapolating mid-term results to long-term implant durability. Investigators, including Young et al. (2020) [22], Gibbons et al. (2025) [26], and Sappey-Marinier et al. (2022) [32], emphasized the need for ongoing surveillance and longer-term follow-up before firm conclusions can be drawn. These expert cautions underscore the importance of tempering enthusiasm for widespread adoption of KA until robust long-term data become available.

A critical finding of this review is the substantial heterogeneity in how KA is defined and implemented across the literature. Distinct approaches were identified, including true KA, restricted KA, anatomical alignment, and functional alignment, each underpinned by different biomechanical principles and alignment targets. The conflation of these fundamentally different techniques in prior reviews has likely obscured important differences in both outcomes and risks.

Our analysis indicates that true KA and restricted KA result in different radiographic alignment profiles and may be associated with differing complication patterns. In addition, functional alignment and anatomical alignment represent separate alignment philosophies and should not be pooled analytically with KA. These findings underscore the need for future studies to clearly define and consistently report alignment techniques, thereby enabling accurate comparison and meaningful synthesis of results.

This review has several notable strengths. It was conducted using a systematic methodology in accordance with PRISMA 2020 guidelines, with independent dual screening and data extraction to minimize selection and extraction bias. A key methodological strength was the explicit differentiation between true KA and related alignment philosophies, allowing for a more nuanced and accurate interpretation of outcomes. In addition, a comprehensive risk of bias assessment was performed, with the implications of study quality clearly discussed. Given the marked heterogeneity across studies, a qualitative synthesis was appropriately undertaken, avoiding inappropriate meta-analytic pooling. Finally, the review presents a balanced appraisal of the evidence, acknowledging both the potential benefits of KA and the unresolved concerns surrounding its widespread adoption.

Several limitations should also be acknowledged. Considerable heterogeneity existed in outcome measures, implant designs, surgical techniques, and follow-up durations, precluding formal meta-analysis. Long-term survivorship data beyond 10 years remain limited, and the predominance of studies conducted in high-volume academic centers may restrict generalizability to routine clinical practice. There is also a potential risk of publication bias favoring positive findings with KA. Furthermore, data on clinically relevant subgroups, such as stratification by alignment phenotype, implant design, or severity of preoperative deformity, were insufficient. Objective gait analysis was performed in only three studies, all with relatively small sample sizes, limiting the strength of biomechanical conclusions.

From a clinical practice perspective, several implications can be drawn from the current evidence. At the mid-term follow-up of two to ten years, KA produces functional outcomes that are generally equivalent to those achieved with MA, with some studies reporting statistically significant improvements in PROMs that may not consistently reach clinical importance. Potential benefits of KA appear most evident in patients with preoperative varus alignment phenotypes, whereas neutral phenotypes show no consistent advantage. Implant selection is an important consideration, as caution is warranted when combining non-neutral alignment strategies with posterior-stabilized implants, given the reported increase in aseptic loosening with restricted KA in one study. Kinematic alignment is also a technique-sensitive approach that may require substantial surgical experience to reproduce reported outcomes safely. In light of limited long-term data, patients undergoing KA should be enrolled in registry-based or structured surveillance programs to enable monitoring of long-term outcomes. Importantly, equipoise remains, as MA continues to be a proven and reliable technique with decades of registry evidence demonstrating excellent long-term survivorship, and routine adoption of KA is not currently justified based on the available evidence.

Future research should aim to address the remaining knowledge gaps. There is a clear need for adequately powered randomized controlled trials with a minimum follow-up of 10 to 15 years, ideally linked to national joint registries. Future studies should incorporate predefined subgroup analyses based on preoperative alignment phenotype, implant design, and the degree of coronal deformity. Standardization of alignment definitions and reporting is essential to enable meaningful comparison and synthesis across studies. Further work is also required to identify patient-specific factors that predict benefit from KA versus MA to evaluate the cost-effectiveness of KA techniques, particularly those requiring advanced imaging or robotic assistance, and to investigate the biomechanical tolerance of specific implant designs to non-neutral alignment. Finally, multicenter studies conducted in community practice settings are needed to assess the reproducibility and generalizability of KA outcomes beyond high-volume academic centers.

## Conclusions

KA achieves mid-term clinical outcomes (2-10 years) that are generally comparable to MA in primary TKA. While some studies report statistically significant improvements in PROMs favoring KA, particularly in OKS, WOMAC, and KSS, these differences often approach but do not consistently exceed MCIDs, and multiple high-quality randomized trials found no clinically meaningful advantage. Radiographic differences consistent with the KA philosophy, including non-neutral coronal alignment and improved joint-line restoration, do not appear to adversely affect mid-term clinical outcomes in most studies, though concerns regarding increased aseptic loosening with restricted KA and posterior-stabilized implants warrant further investigation. Importantly, evidence regarding long-term implant durability beyond 10 years remains limited, and statistical power for survivorship comparisons is insufficient due to low event rates. The available 10-year data from high-quality randomized controlled trials demonstrate equivalent survivorship between techniques, but the critical 10-20-year period remains largely unstudied. Given these limitations, MA remains a proven reference standard, and routine adoption of KA is not currently justified based on available evidence. Surgeons considering KA should carefully select appropriate patients, ensure adequate technical expertise, and enroll patients in long-term surveillance programs. Further long-term studies (≥15 years), standardized biomechanical evaluations, and subgroup analyses by patient phenotype and implant design are required to definitively establish the role of KA in contemporary TKA practice.

## Appendices

Detailed electronic search strategy

Search overview

The electronic literature search was completed on June 13, 2025, and addressed the topic Kinematic Alignment versus Mechanical Alignment in Total Knee Arthroplasty. The search strategy was developed and executed with the assistance of a clinical librarian (Nikki Myall, Clinical Librarian, Maidstone & Tunbridge Wells NHS Trust). No date restrictions were applied, and all databases were searched from inception to the search date.

MEDLINE (Ovid Interface)

Database: Ovid MEDLINE(R) ALL (1946 to June 13, 2025)

The MEDLINE search combined controlled vocabulary (MeSH terms) and free-text keywords to capture concepts relating to total knee arthroplasty, alignment philosophy, and clinical or functional outcomes. The population was identified using the MeSH term Arthroplasty, Replacement, and Knee and relevant free-text synonyms, including TKA, total knee replacement, total knee arthroplasty, TKR, primary knee replacement, and tricompartmental knee replacement. Alignment concepts were captured using the MeSH term Biomechanical Phenomena and proximity-based free-text terms relating to kinematic and mechanical alignment. Outcomes were identified using MeSH terms for treatment outcome, range of motion, and postoperative complications, alongside validated patient-reported outcome measures and survivorship-related keywords.

Search concepts were combined using Boolean operators (AND/OR). Filters were applied to limit results to English-language and human studies. Duplicate records were removed from the database.

Final yield: 1,150 unique records.

Embase (Ovid Interface)

Database: Embase (1974 to 2025, Week 24)

A structured Embase search was performed using EMTREE terms and free-text keywords. The population concept included the EMTREE term total knee arthroplasty and equivalent synonyms. Alignment was captured using EMTREE terms for biomechanics and kinematics, in combination with proximity-based free-text terms for kinematic and mechanical alignment. Outcomes were identified using EMTREE terms for treatment outcome, range of motion, and postoperative complications, supplemented by validated outcome measures and survivorship-related terms.

Results were limited to English-language and human studies. Records indexed in MEDLINE were excluded to avoid duplication. Automated and manual deduplication was performed.

Final yield: 852 unique records (after exclusion of MEDLINE overlap).

Cochrane Central Register of Controlled Trials (CENTRAL)

Database: Cochrane CENTRAL (Issue 6 of June 12, 2025)

The CENTRAL search strategy combined MeSH headings and free-text terms relating to knee arthroplasty, alignment concepts, and clinical outcomes. Knee arthroplasty terms were combined with alignment-related keywords using proximity operators, and outcome terms included validated patient-reported outcome measures, range of motion, survivorship, and revision. The search was limited to randomized controlled trials.

Final yield: 296 records.

Google Scholar

A supplementary search was conducted in Google Scholar using keyword combinations related to total knee arthroplasty, kinematic and mechanical alignment, and mid-term or functional outcomes. Results were sorted by relevance using the default Google Scholar algorithm.

The first 600 records were screened by title. Non-English publications and duplicate citations already retrieved from bibliographic databases were excluded. Potentially relevant articles underwent full-text assessment against predefined eligibility criteria.

Approximately 587 records were manually reviewed. No date restrictions were applied. Due to the proprietary nature of Google Scholar’s ranking algorithms, exact reproducibility is limited; therefore, Google Scholar was used as a supplementary source only, and all included studies were verified in primary databases.

Deduplication Process

All retrieved records were imported into EndNote X20 (Clarivate Analytics) for deduplication. Automated duplicate detection was performed using EndNote’s algorithm based on author, title, year, and DOI matching. A total of 156 potential duplicates were manually reviewed and verified. Cross-database duplicates were removed.

Total records retrieved: 2,885

Final unique records after deduplication: 1,662

Search Limits and Eligibility Considerations

Search limits were applied as follows: only English-language studies and human studies were included. No date restrictions were applied in any database, and all studies from database inception to 13 June 2025 were eligible. No study-design filters were applied at the search stage; eligibility by study design was determined during title/abstract and full-text screening in accordance with predefined inclusion and exclusion criteria.

## References

[1] Abdul N, Dixon D, Walker A (2015). Fibrosis is a common outcome following total knee arthroplasty. Sci Rep.

[2] Ren Y, Cao S, Wu J, Weng X, Feng B (2019). Efficacy and reliability of active robotic-assisted total knee arthroplasty compared with conventional total knee arthroplasty: a systematic review and meta-analysis. Postgrad Med J.

[3] Beckers G, Meneghini RM, Hirschmann MT, Kostretzis L, Kiss MO, Vendittoli PA (2024). Ten flaws of systematic mechanical alignment total knee arthroplasty. J Arthroplasty.

[4] Fang DM, Ritter MA, Davis KE (2009). Coronal alignment in total knee arthroplasty: just how important is it?. J Arthroplasty.

[5] Parratte S, Pagnano MW, Trousdale RT, Berry DJ (2010). Effect of postoperative mechanical axis alignment on the fifteen-year survival of modern, cemented total knee replacements. J Bone Joint Surg Am.

[6] Nedopil AJ, Howell SM, Hull ML (2020). Deviations in femoral joint lines using calipered kinematically aligned TKA from virtually planned joint lines are small and do not affect clinical outcomes. Knee Surg Sports Traumatol Arthrosc.

[7] Baker PN, van der Meulen JH, Lewsey J, Gregg PJ (2007). The role of pain and function in determining patient satisfaction after total knee replacement. Data from the National Joint Registry for England and Wales. J Bone Joint Surg Br.

[8] Beswick AD, Wylde V, Gooberman-Hill R, Blom A, Dieppe P (2012). What proportion of patients report long-term pain after total hip or knee replacement for osteoarthritis? A systematic review of prospective studies in unselected patients. BMJ Open.

[9] Lustig S, Sappey-Marinier E, Fary C, Servien E, Parratte S, Batailler C (2021). Personalized alignment in total knee arthroplasty: current concepts. SICOT J.

[10] Howell SM, Papadopoulos S, Kuznik KT, Hull ML (2013). Accurate alignment and high function after kinematically aligned TKA performed with generic instruments. Knee Surg Sports Traumatol Arthrosc.

[11] Dossett HG, Swartz GJ, Estrada NA, LeFevre GW, Kwasman BG (2012). Kinematically versus mechanically aligned total knee arthroplasty. Orthopedics.

[12] Liu B, Feng C, Tu C (2022). Kinematic alignment versus mechanical alignment in primary total knee arthroplasty: an updated meta-analysis of randomized controlled trials. J Orthop Surg Res.

[13] Davis KR, Soti V (2024). Effectiveness of kinematic alignment-total knee arthroplasty in treating preoperative varus and valgus deformities in patients with knee osteoarthritis. Cureus.

[14] Nagai K, Niki Y, Kobayashi S, Harato K, Nagura T, Matsumoto M, Nakamura M (2021). Radiographic evaluation of patellofemoral alignment in kinematically aligned total knee arthroplasty: a comparative study with mechanically aligned total knee arthroplasty. J Orthop Sci.

[15] Page MJ, McKenzie JE, Bossuyt PM (2021). The PRISMA 2020 statement: an updated guideline for reporting systematic reviews. BMJ.

[16] Ouzzani M, Hammady H, Fedorowicz Z, Elmagarmid A (2016). Rayyan-a web and mobile app for systematic reviews. Syst Rev.

[17] Seon JK, Song EK, Lee DH (2017). Comparison of outcome and gait analysis after robotic total knee arthroplasty between mechanical and kinematic knee alignment methods with average 8 years follow up. Arthroscopy.

[18] Dossett HG, Estrada NA, Swartz GJ, LeFevre GW, Kwasman BG (2014). A randomised controlled trial of kinematically and mechanically aligned total knee replacements: two-year clinical results. Bone Joint J.

[19] Young SW, Walker M, Bayan A (2017). No difference in 2-year functional outcomes using kinematic versus mechanical alignment in total knee arthroplasty: a randomized controlled clinical trial. Arthroscopy.

[20] Young SW, Walker ML, Bayan A, Briant-Evans T, Pavlou P, Farrington B (2017). The Chitranjan S. Ranawat Award : no difference in 2-year functional outcomes using kinematic versus mechanical alignment in TKA: a randomized controlled clinical trial. Clin Orthop Relat Res.

[21] McEwen PJ, Dlaska CE, Jovanovic IA, Doma K, Brandon BJ (2020). Computer-assisted kinematic and mechanical axis total knee arthroplasty: a prospective randomized controlled trial of bilateral simultaneous surgery. J Arthroplasty.

[22] Young SW, Sullivan NP, Walker ML, Holland S, Bayan A, Farrington B (2020). No difference in 5-year clinical or radiographic outcomes between kinematic and mechanical alignment in TKA: a randomized controlled trial. Clin Orthop Relat Res.

[23] Kim TW, Lee JI, Choi HG, Yoo HJ, Kim KT, Lee YS (2022). Comparison of the radiologic, morphometric, and clinical outcomes between kinematically and mechanically aligned total knee arthroplasty: a propensity matching study. J Knee Surg.

[24] Dossett HG, Arthur JR, Makovicka JL, Mara KC, Bingham JS, Clarke HD, Spangehl MJ (2023). A randomized controlled trial of kinematically and mechanically aligned total knee arthroplasties: long-term follow-up. J Arthroplasty.

[25] Sarzaeem MM, Movahedinia M, Mirahmadi A, Abolghasemian M, Tavakoli M, Amouzadeh Omrani F (2024). Kinematic alignment technique outperforms mechanical alignment in simultaneous bilateral total knee arthroplasty: a randomized controlled trial. J Arthroplasty.

[26] Gibbons JP, Zeng N, Bayan A, Walker ML, Farrington B, Young SW (2025). No difference in 10-year clinical or radiographic outcomes between kinematic and mechanical alignment in TKA: a randomized trial. Clin Orthop Relat Res.

[27] Young SW, Tay ML, Kawaguchi K, van Rooyen R, Walker ML, Farrington WJ, Bayan A (2025). The John N. Insall Award: functional versus mechanical alignment in total knee arthroplasty: a randomized controlled trial. J Arthroplasty.

[28] Koh DT, Woo YL, Yew AK, Yeo SJ (2021). Kinematic aligned femoral rotation leads to greater patella tilt but similar clinical outcomes when compared to traditional femoral component rotation in total knee arthroplasty. A propensity score matched study. Knee Surg Sports Traumatol Arthrosc.

[29] Nakagawa Y, Koga H, Sekiya I, Hasegawa S, Katagiri H, Watanabe T (2025). Equivalent clinical outcomes between anatomical alignment versus mechanical alignment of simultaneous bilateral total knee arthroplasty using a posterior-stabilized prosthesis during an average follow-up of five years: a prospective randomized clinical trial. J Arthroplasty.

[30] Kobayashi T, Kawaguchi K, Goto K, Suzuki H, Otsu M, Michishita K (2024). Functional knee phenotypes: a helpful classification tool for visualizing potential femoral varus in restricted kinematic alignment total knee arthroplasty in Japan. Knee Surg Sports Traumatol Arthrosc.

[31] Ettinger M, Tuecking LR, Savov P, Windhagen H (2024). Higher satisfaction and function scores in restricted kinematic alignment versus mechanical alignment with medial pivot design total knee arthroplasty: a prospective randomised controlled trial. Knee Surg Sports Traumatol Arthrosc.

[32] Sappey-Marinier E, Shatrov J, Batailler C, Schmidt A, Servien E, Marchetti E, Lustig S (2022). Restricted kinematic alignment may be associated with increased risk of aseptic loosening for posterior-stabilized TKA: a case-control study. Knee Surg Sports Traumatol Arthrosc.

